# Investigation of mild steel corrosion inhibition in acidic media by *Viola* extract based on bulk and nanometer size

**DOI:** 10.1038/s41598-024-66434-x

**Published:** 2024-07-04

**Authors:** Razieh Naghizade, Ghazal Sadat Sajadi, Abofazel Khosravi Mashizi, Zahra Golshani, Mahnaz Amiri, Seyed Mohammad Ali Hosseini

**Affiliations:** 1 Department of Chemistry, Shahid Bahonar University of Kerman, P.O. Box, Kerman, 76169-14111 Iran; 2 Department of Hematology and Laboratory Sciences, Faculty of Allied Medicine, Kerman University of Medical Sciences, Kerman, Iran

**Keywords:** Mild steel, *Viola*, Nano size, Green inhibitor, Electrochemical impedance spectroscopy, Polarization technique, Chemistry, Nanoscience and technology

## Abstract

In the present work, the inhibition performance of *Viola* extract based on bulk and nano size as a green corrosion inhibitor on mild steel in 0.5 M phosphoric acid and 1M hydrochloric acid solutions is investigated using different techniques (potentiodynamic polarization, electrochemical impedance spectroscopy (EIS) and Optical microscopy). The gained results demonstrated that various concentrations of *Viola* Extract (bulk and nano) inhibited the corrosion of the alloy in both of the acid solutions. The temperature impact on corrosion rate without/with this extract was examined. Certain thermodynamic parameters were determined based on the temperature impact on inhibition and corrosion processes. The adsorption mechanism of the extract on the alloy was explored using the Langmuir adsorption isotherm. A mixed mode of adsorption was observed, wherein the nano-sized extract in 1.0 M HCl predominantly underwent chemisorption, while the bulk-sized extract in 1.0 M HCl and both bulk and nano-sized extracts in 0.5 M H_3_PO_4_ were primarily subjected to physisorption. Scanning electron microscopy (SEM) and Optical microscopy analyses were employed to scrutinize alloys’ surface morphology.

## Introduction

Metals and alloys are used extensively by humans due to their good physical and electrical qualities^1^. Metal corrosion is a big problem that happens all over the world in many industries where things are made^2^. Mild steels are widely used in several sectores like gas and oil. It is commonly utilized for processes like acid pickling, chemical cleaning, acid descaling, and oil well acidizing^3^. However, these materials have a big problem because they don't resist corrosion well in acidic liquids^4^. Phosphoric acid finds application in electrolytic polishing, oxide film removal, phosphating, passivation, and fertilizer production. Despite not being classified as a strong acid; it significantly harms on iron^5^. Hydrochloric acid is extensively utilized across various industries for purposes such as oil-well acidification, acid cleaning, pickling, and descaling processes^6^.

The corrosion of metals can be prevented or slowed down by using different techniques. These techniques include applying protective coatings, using electrical protection, choosing the right materials, and using inhibitors^7–10^. Inhibitors are chemical substance that can prevent metal surfaces from corroding when utilized in small scale in corrosive media^11,12^. Using corrosion inhibitors is a low cost method to decrease the destroying rate, defend metal surfaces from corrosion, and eventually shield equipment in tough places^13^.

Inhibitors help to control or stop the interaction at the intersection where the aggressive liquid and the metal meet. This affects how chemical reactions occur on the metal surface by adsorption on the surface^14^. Corrosion inhibitors are widely used in many industries to slow down the process of metal products being damaged when exposed to a challenging environment. The efficiency of corrosion inhibitors depends on their power to stick to metal surfaces^15^. The ability of corrosion inhibitors to break down naturally, the amount that accumulates over time, and how harmful they are have all been questioned recently. Researchers are worried about three important things about inhibitors: their safety, pollution of the environment, and cost. They are trying to find inhibitors that are safe, do not cause pollution, and are not expensive^16^.

In the recent years, many people have become interested in green chemistry. the principle of “green chemistry” refers to efforts toward establishing a comprehensive approach to chemical risk management. This concept is based on the ideas of sustainability, reducing environmental consequences, and preserving natural resources for the following centuries^17^. Researchers are also finding ways to make less waste and avoid dangerous substances in the products. They are paying a lot of attention to green inhibitors because they can help prevent corrosion and can be renewed, are environmentally friendly, can be broken down by nature, and are safe^18^. So, Scientists are trying to find green ways to stop corrosion in effective but less harmful ways^19^.

In recent times, researchers have turned their attention to plant extracts as a means to develop alternative, cost-effective, and environmentally friendly corrosion inhibitors^20–26^. Arthur et al.^27^ surveyed the inhibitory impact of *Acalypha chamaedrifolia* leaf extract on mild steel corrosion in hydrochloric acid. Zaher et al.^28^ examined *Ammi visnaga* L. extract as a corrosion inhibitor for mild steel in HCl solution, achieving an inhibition efficiency of 84%. Shahmoradi et al.^29^ demonstrated the efficacy of quince seed extract as a mild steel corrosion inhibitor in 1 M HCl solution, with a corrosion efficiency of 95% at an extract concentration of 800 ppm. Theoretical and empirical surveys identified Inula viscosa extract as an efficient inhibitor for mild steel corrosion in 1 M HCl, providing up to 92% efficiency^30^. Eucalyptus plant leaf extract proved to be a proficient natural corrosion inhibitor in 0.5 M H3PO4 and 0.5 M H_2_SO_4_ solutions for mild steel^31^. Aqueous black mustard seeds were investigated as a sustainable green inhibitor in 2 M H_2_SO_4_ for mild steel corrosion^32^. Boudalia et al.^33^ explored the corrosion inhibition impact of Artemisia essential oil for carbon steel corrosion control in 1 M H_3_PO_4_ solution. Aisha Hussain Al-Moubaraki et al.^34^ examined the inhibitory effects of Natural Plant Extracts of Zingiber zerumbet (ZZAE), Fraxinus excelsior (FEAE), and Isatis tinctoria on mild steel corrosion in phosphoric acid.

Nanostructured materials have been studied considerable because of their broad range of prominent applications because nanostructures exhibit novel size-dependent properties, such as chemical properties, that extensively differ from their bulk materials, that exhibit great potential in the novel fields. The increasing trend of nano-scale production particularly for encapsulating herb and spice extract has been reported to have some advantages such as improving bioavailability, biological activity and stability as well as controlling the release of bioactive compounds^35^.

**Figure 1:**
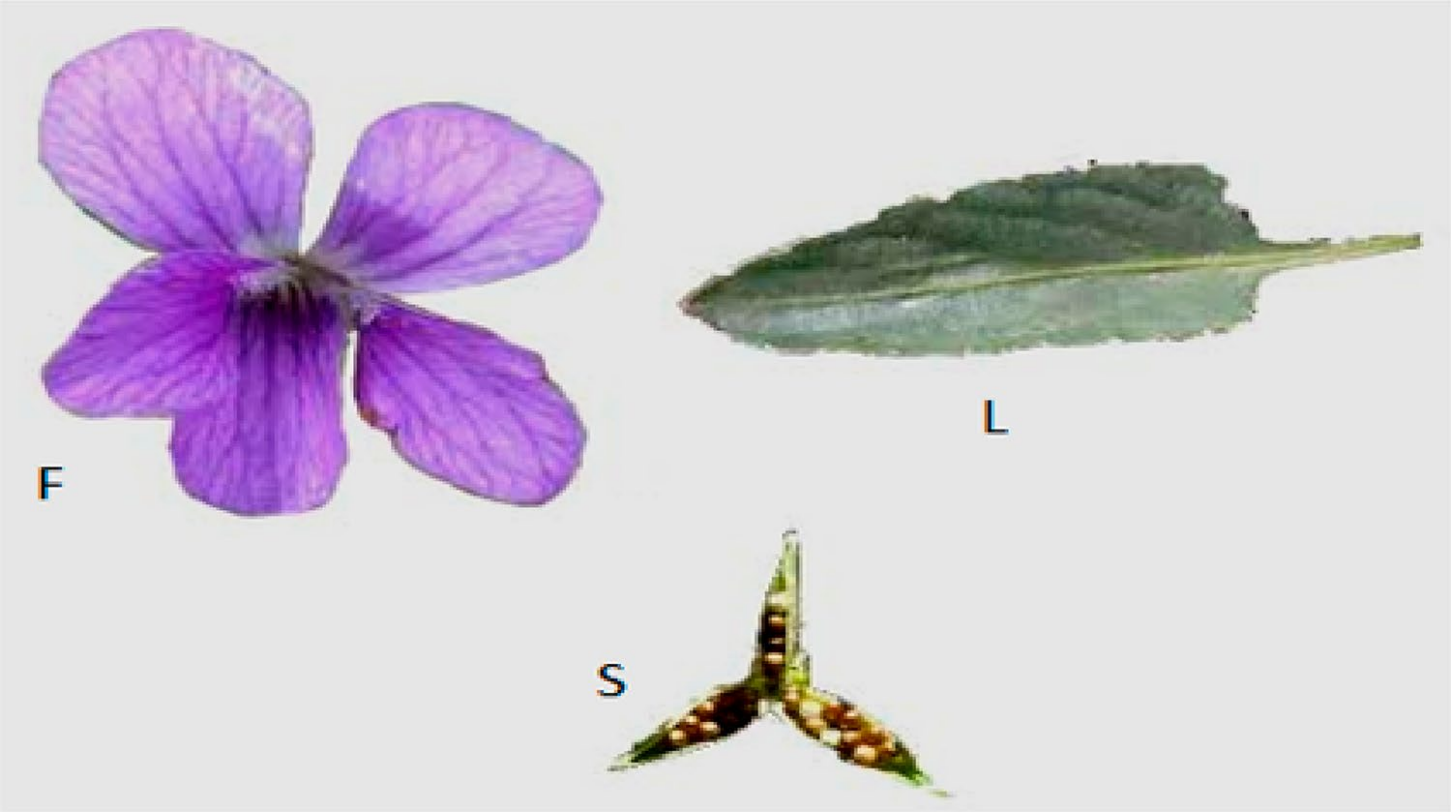
Different parts of *Viola plant,* L., leaf; F., flower; S, seed.

**Figure 2:**
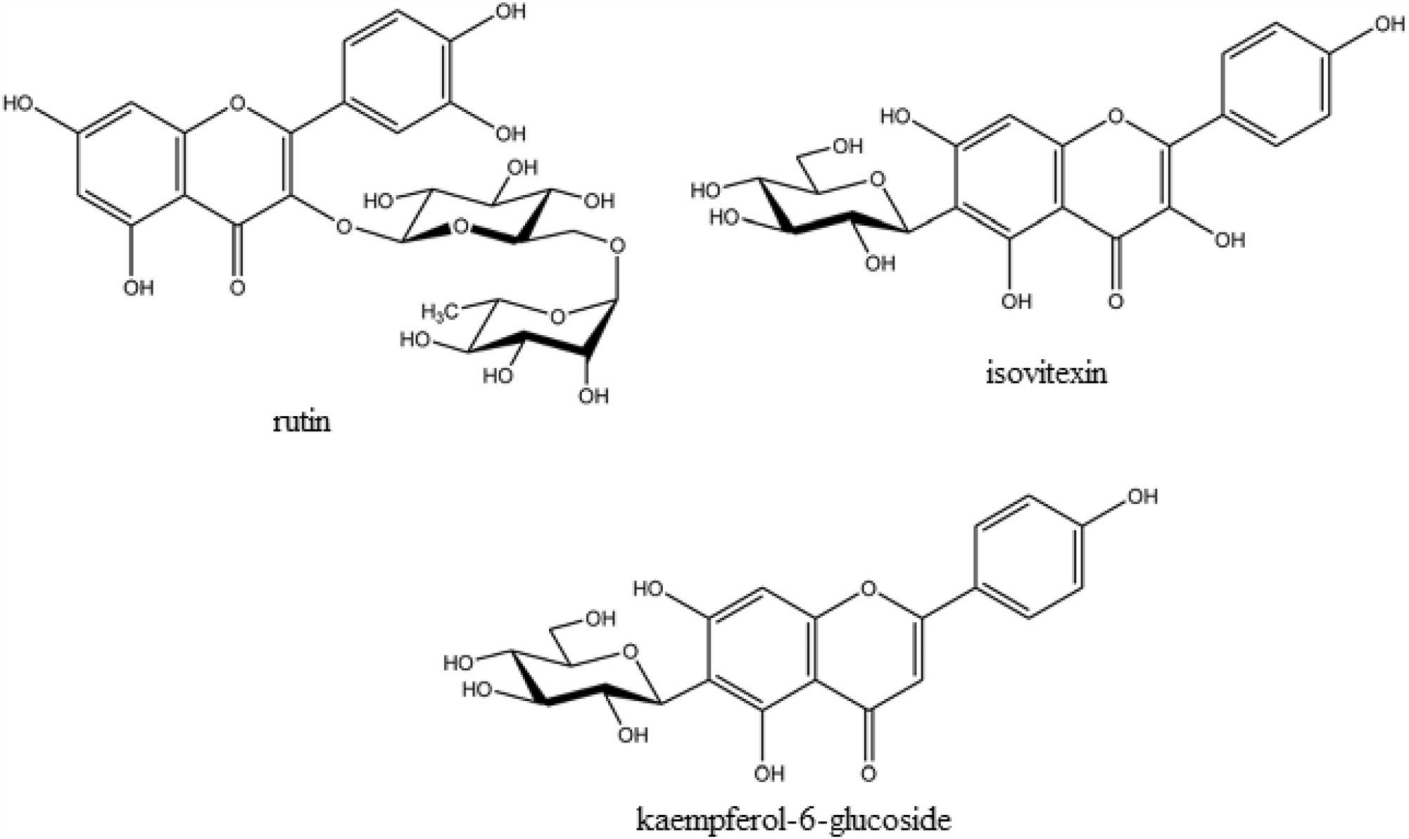
Structures of *Viola*.

The *Viola* genus, belonging to the *Viola* cease family, is one of the largest genera, encompassing 525–600 species distributed across most regions of the world and categorized into 14 sections and numerous sub-sections^36^. Renowned for its medical features, including anthelminthic, antioxidant, anti-inflammatory, analgesic, and antidepressant effects, it has been documented for treating different neurological disorders^37^. This perennial herb typically reaches heights of 8–20 cm, characterized by elongated, slender arrow-shaped leaves that often widen from the base, lacking stems and measuring approximately 2.4 inch (6 cm) in length with a V-shaped sinus base. Different parts of the *Viola plant are shown in* Fig. 1^37^. The compounds identified in *Viola* species include rutin, isovitexin, and kaempferol-6-glucoside (Fig. 2)^38^.

*Viola* Extract is a great option to use as an eco-friendly green inhibitor because it doesn’t have any dangerous metals or harmful substances. It also has some advantages such as being easy to get to, the friendly environment and high accessibility. Lately, it has been started applying as green corrosion inhibitors instead of organic corrosion inhibitors. In this study, investigated how green corrosion inhibitors can protect mild steel (st-37) from corrosion. Specifically looked at the inhibitory effect of *Viola* extract at both in bulk, and nanoscale when exposed to acidic environments. Besides, two methods are utilized, potentiodynamic polarization (PP) and electrochemical impedance spectroscopy (EIS), to investigate the corrosion process. As far as we are aware, this extract both in bulk, and nanoscale, is being investigated for the first time for its corrosion inhibition effect on mild steel.

## Experimental details

### Materials

Materials were ready to use without any additional cleaning or purifying. These materials were obtained from Arshanzist Youtab Company. To make the liquids needed for the experiment, utilized some chemicals: phosphoric acid, hydrochloric acid, ethyl alcohol, methanol, and distilled water.

#### Preparation of work electrodes to electrochemical measurements

The work electrodes for the corrosion measurements were prepared of mild steel. The samples had a size of 1 square centimeter for all tests. The abaraded side of the metal sheets were made shiny by using different types of sandpaper (100, 400, 1000, and 2500 grit).

#### *Viola* extract preparation

The *Viola* plant contains eaves, flowers and seeds that were bought from the markets in Iran. To get rid of dust, the plants were washed and then they were left in a cool, shady area inside a room until they were dried. At normal room temperature and without any light, 100 g of dried *Viola* plants were put in distilled water for 72 h. The extra liquid was removed by heating it in a special container at 40 °C, after removing any solid particles. The leftover material weighed 2. 0 g.

#### Declaration for the usage of plant materials

We declare that in this research, we did not use or not to use any plants (either cultivated or wild) irrespective of any location. Experimental research and field study in this study has complied with the IUCN Policy Statement on Research Involving Species at Risk of Extinction. The use of plants in the present study complies with international, national and/or institutional guidelines.

#### Preparation of Nanosized *Viola* extract

*Viola* nano micelle was produced using heated absolute ethanol and isopropanol 400 as solvent, Tween 80 and sodium caseinate dissolved in distilled water as an emulsifier by dissolution method. Firstly, the solvents (absolute ethanol and isopropanol 400) were heated to 40–42 °C. 0.08 g of the emulsifiers (Tween 80 and sodium caseinate 1:1) and the specified amount of *Viola* extract (2 g) were dissolved in deionized water and heated to reach the solvent’s temperature. The solvent phase was added slowly to the aqueous phase containing *Viola* and mixed for 50 s by a homogenizer at 12,800 rpm to create *Viola* nanomicelles. The produced sample was stored in a closed container in the refrigerator for the next steps.

#### Solutions preparation

The electrolytes were made by mixing distilled water with concentrated chemicals. The first liquid was prepared by mixing analytical grade H_3_PO_4_ from Merck with distilled water to make a 0. 5 M H_3_PO_4_ solution. The second liquid was made by mixing concentrated HCl with distilled water to make a 1. 0 M HCl solution. Before each exploration, the test solution was arranged by blending the *Viola* extract with the corrosive medium. Two experiments were conducted to make sure the results could be repeated. The concentrations of the extract in the solutions were 100, 150, 200, and 250 ppm for 0. 5 M H_3_PO_4_ solution and 1. 0 M HCl solution, the concentrations were 75, 100, 125, and 150 ppm.

### Procedures

#### Electrochemical evaluations

The reference, counter, and working electrodes were provided by three-electrode cells that contained Ag/AgCl, Pt electrodes and mild steel, respectively. The Tafel polarization curves were plotted by setting the rate of the polarization scan at 1 mV/s. The potentiodynamic polarization and EIS tests were conducted using the potential and frequency ranges of − 200 to + 200 mV versus Ag/AgCl and 100 mHz to 100 kHz, respectively. The potential stability over 30 min before each polarization and impedance test under temperature conditions of 25 ± 1 °C. Finally, NOVA 1.11 software with URL link https://www.advanceduninstaller.com/Nova-1_11-7fa4b72b3ea3964ce9fbae4e0a289ef6-application.htm was employed to analyze the obtained curves.

#### Temperature impact

The potentiodynamic polarization technique was utilized to study the temperature impact on the mild steel corrosion rate in 0.5 M H_3_PO_4_ and 1 M HCl when different *Viola* extract concentrations were absent and present on bulk while also examining the nano-size at a temperature range of 25–45 ± 1 °C.

#### Examination of surface morphology

The working electrode’s surface morphology was examined by optical images after alloy plunging over 24-h in both solutions under room temperature when the optimum extract concentrations were absent and present with bulk and nanometer size.

## Results and discussion

### SEM, DLS analysis

**Figure 3:**
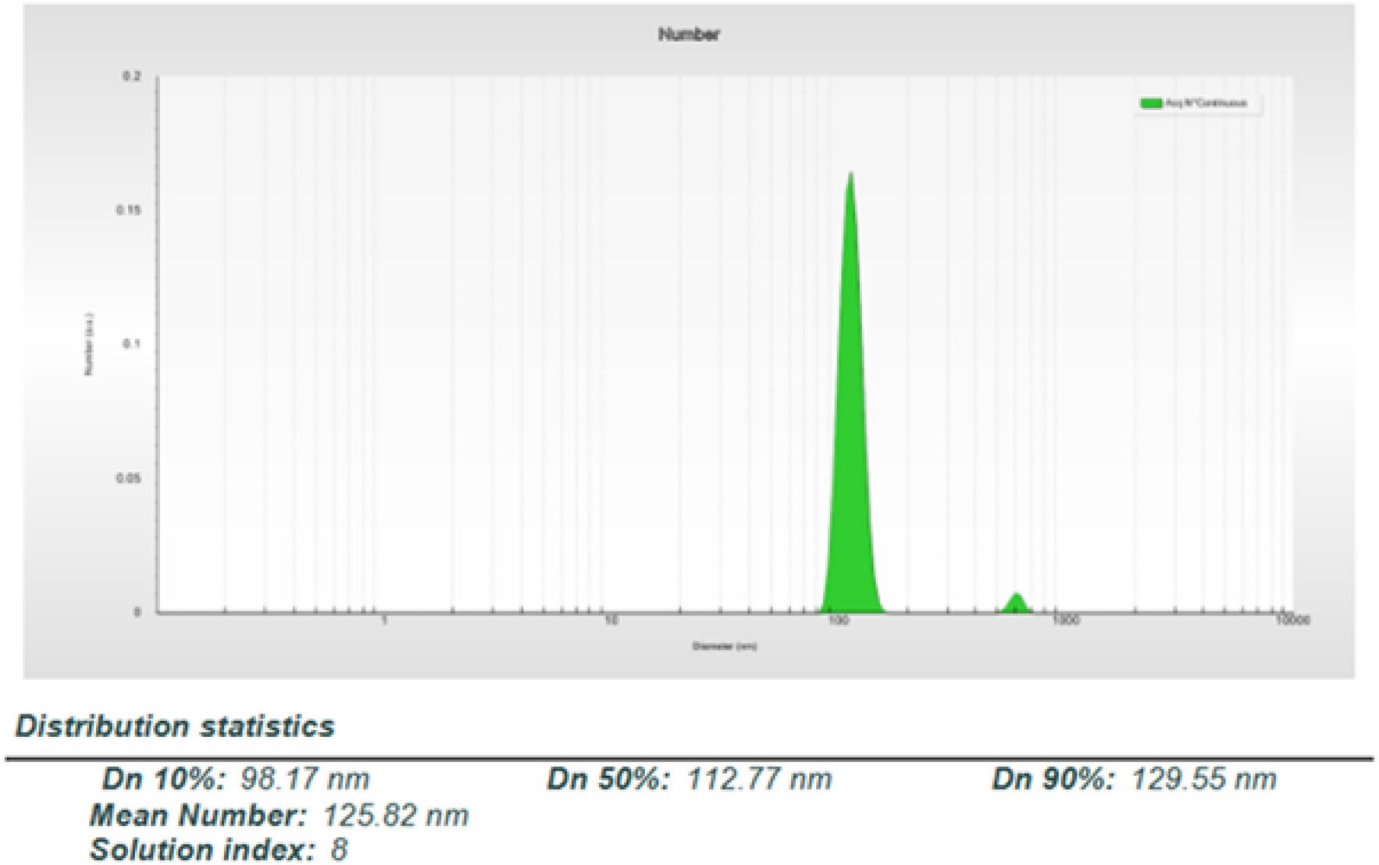
The mean size of produced nanoparticles recorded by the nanosizer equipment (DLS technique).

The histogram of the SBL nanosizer of NPs in Fig. 3 shows that the mean diameter of particle size is located in nanometer range but not really under 100 nm. The reported results showed that NPs had a narrow size distribution and a homogenous dispersity.

**Figure 4:**
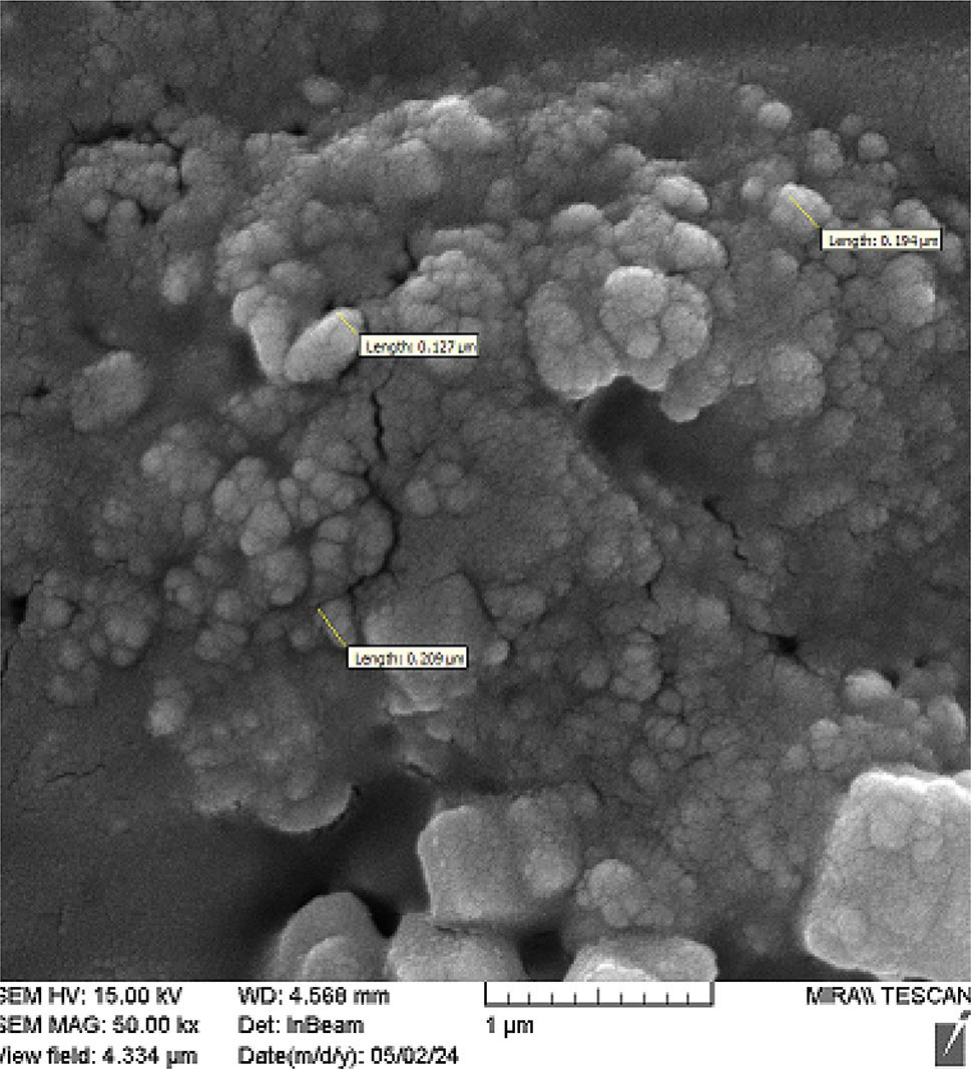
The images of nanosized extract using Scanning electron microscopy (SEM).

The morphology of nanosized extract was observed by SEM. Different magnifications of the images are shown in Fig. 4. The spherical particles have grown and some agglomeration was detected.

### Corrosion inhibition study

In the first step, the working electrode is placed in two different solutions, one with HCl and one with H_3_PO_4_ with and without *Viola* extract, both in bulk and nanometer quantities. The electrode was left in the solutions for 30 min, and observed that the open-circuit potential (OCP) became stable. Then, the electrochemical measurements were carried out. The behavior of corrosion of st-37 in 1.0 M HCl and 0.5 M H_3_PO_4_ media was characterized by EIS, and PP techniques beneath distinctive concentrations of *Viola* extract based on bulk and nano quantities.

### Potentiodynamic polarization

**Figure 5:**
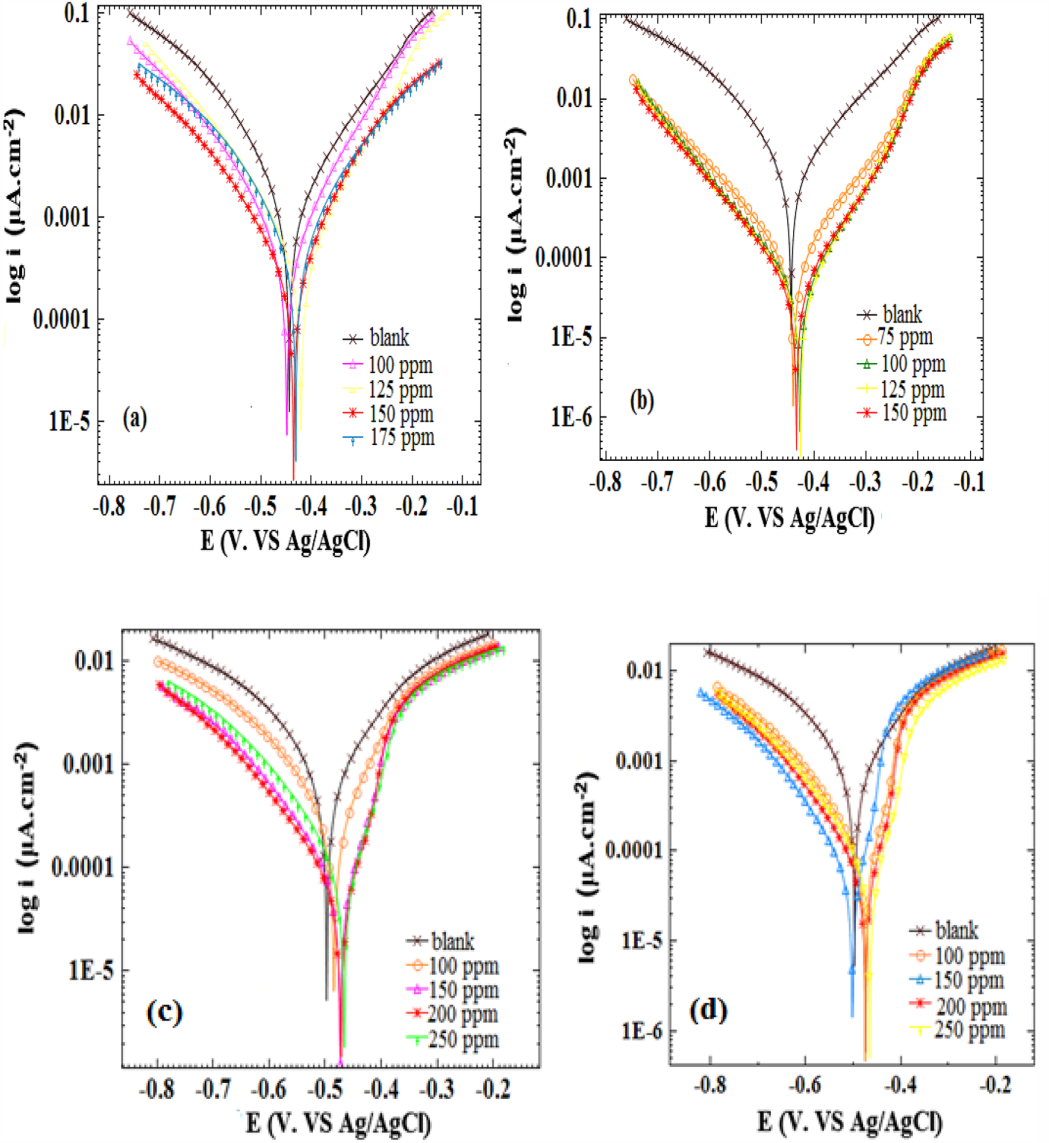
Polarization curves for st-37 in 1.0 M HCl (**a**) based on bulk, and (**b**) nano size of *Viola*, and in 0.5 M H_3_PO_4_ (**c**) based on bulk, and (**d**) nano size of *Viola* at 25 $\pm$ 1 °C.

The inhibition concentration impact on the alloy corrosion was examined through Potentiodynamic polarization measurements, considering 0.5 M H_3_PO_4_ and 1.0 M HCl conditions when different *Viola* extracts (bulk, and nanosize) concentrations were absent and present. As shown by the experimental results, there was a significant decrease in the corrosion current density as the inhibitor concentration increased up to 150 ppm in 1.0 M HCl, and up to 200 ppm, for 0.5 M H3PO4 including bulk, and nano size of the extract. The resulting plots for acidic conditions are illustrated in Fig. 5. The electrochemical parameters of corrosion potential (E_corr_), Corrosion current density (j_corr_), cathodic Tafel slope (βc), Anodic Tafel slope (βa), surface coating (θ), inhibitory percentage (IE %) resulting from the polarization investigations are presented in Tables 1 and 2.

**Table 1:** Corrosion parameters derived from polarization curves for st-37 in HCl solution with (a) inhibitor, (b) nano inhibitor.

C/ppm	j_corr_/μA cm^−2^	− E_corr_/mV	− β_c_/mV dacade^−1^	β_a_/mV dacade^−1^	θ	IE%
(a) Viola/HCl(1M)						
blank	847	444	114	95	–	–.
100	323	449	96	99	0.62	62
125	277	421	103	96	0.68	68
150	216	436	96	117	0.75	75
175	296	431	91	96	0.65	65
(b)Viola(nano/HCl(1M)						
blank	847	444	114	95	–	–
75	83	440	108	126	0.9	90
100	35	428	114	92	0.95	95
125	35	426	88	124	0.95	95
150	34	434	109	95	0.96	96

**Table 2:** Corrosion parameters derived from polarization curves for st-37 in H_3_PO_4_ solution with (a) inhibitor, (b) nano inhibitor.

C/ppm	j_corr_/μA cm^−2^	− E_corr_/mV	− β_c_/mV dacade^−1^	β_a_/mV dacade^−1^	θ	IE%
(a) Viola/H_3_PO_4_ (0.5M)						
blank	424	498	106	100	–	–
100	155	485	89	76	0.63	63
150	34	474	51	75	0.92	92
200	28	473	74	50	0.93	93
250	48	467	78	56	0.88	88
(b)Viola(nano/H_3_PO_4_ (0.5M)						
blank	424	498	106	100	–	–
100	64	475	102	55	0.84	84
150	26	502	74	42	0.93	93
200	25	475	69	47	0.94	94
250	33	465	53	74	0.91	91

The equation below calculates the inhibitory efficiency^39^:

1 $$\text{IE}\%=\left(\frac{{\text{j}}_{\text{corr}}-{\text{j}}_{\text{inh}}}{{\text{j}}_{\text{corr}}}\right) \times 100$$

In which, j_corr_ and j_inh_ indicate the current density without and with inhibitor, respectively, obtained by extrapolation of polarization plots Tefal lines. As shown, there are a decrease in the corrosion current density (from 847 to 216 μA/cm^2^) for the alloy in blank HCl solution and solution containing bulk inhibitor and the decrease in the corrosion current density (from 847 to 34 μA/cm^2^) for the alloy in blank HCl solution and solution containing nanosize inhibitor. There are increase in IE% to 75%, and 96% for HCl solutions with bulk, and nano size of the extract. Also, there are decrease in the corrosion current density (from 424 to 28 μA/cm^2^) for the alloy in blank H_3_PO_4_ solution and solution containing bulk inhibitor and the decrease in the corrosion current density (from 424 to 25 μA/cm^2^) for the alloy in blank H_3_PO_4_ solution and solution containing nanosize inhibitor. There are increase in IE% to 93%, and 94% for H_3_PO_4_ solutions with bulk, and nano size of the extract.Thus, the bulk and nanosize extract has an inhibitory impact, forming a protective layer when absorbed on the metal surface. Given that higher concentrations lead to higher inhibitory efficiency; it is possible to obtain this protective layer at higher considerations^40^.

The corrosion potential does not typically undergo significant changes by the mixed inhibitors. Based on the experimental results, there are no substantial changes in the corrosion potential at higher inhibitor concentrations under two-acid conditions, highlighting the inhibitor’s performance as a mixed type^41^. Moreover, variances in the βa and βc values in comparison with blank solutions indicate the protective effects of inhibitors against the corrosion process via adsorption of inhibitor molecules on cathodic and anodic sites. The inclusion of inhibitors brings about noticeable alterations in the cathodic and anodic segments of Tafel plots in both HCl and H_3_PO_4_ solutions. Consequently, it is categorized as a mixed-type inhibitor. On the other hand, the maximum shift in *E*_corr_ value was positive/negative side 18, and 23 mV, respectively, for HCl and H_3_PO_4_ solutions. Based on the literature, corrosion potential shifts of <$\pm$ 85 mV concerning the blank solution leads to the mixed-type function of the inhibitor, confirming the mixed type of this inhibitor^42^.

### Electrochemical impedance spectroscopy

**Figure 6:**
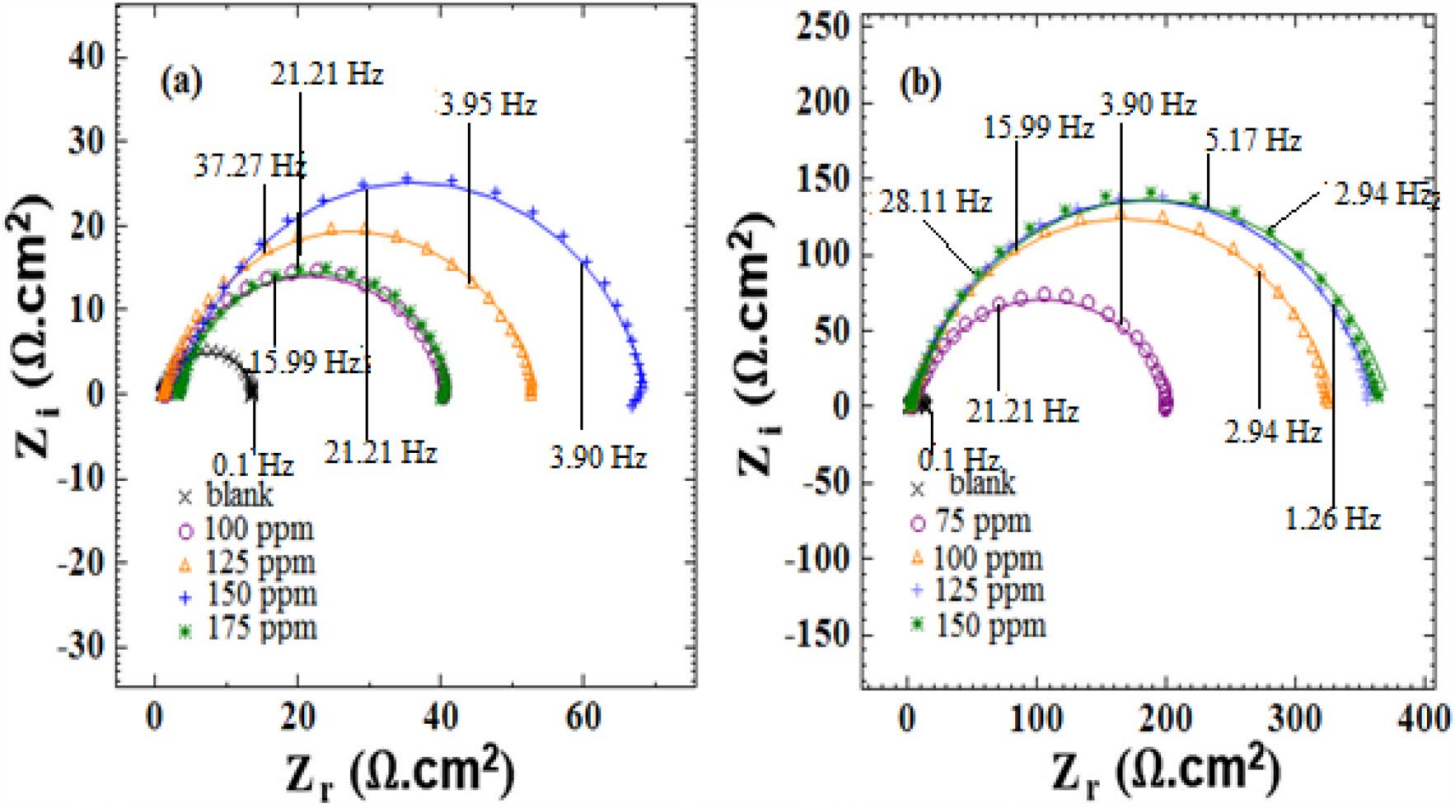
The (**a**, **b**) Nyquist plots for st-37 with different concentrations of *Viola* based on bulk, and nano size, in 1M HCl.

**Figure 7:**
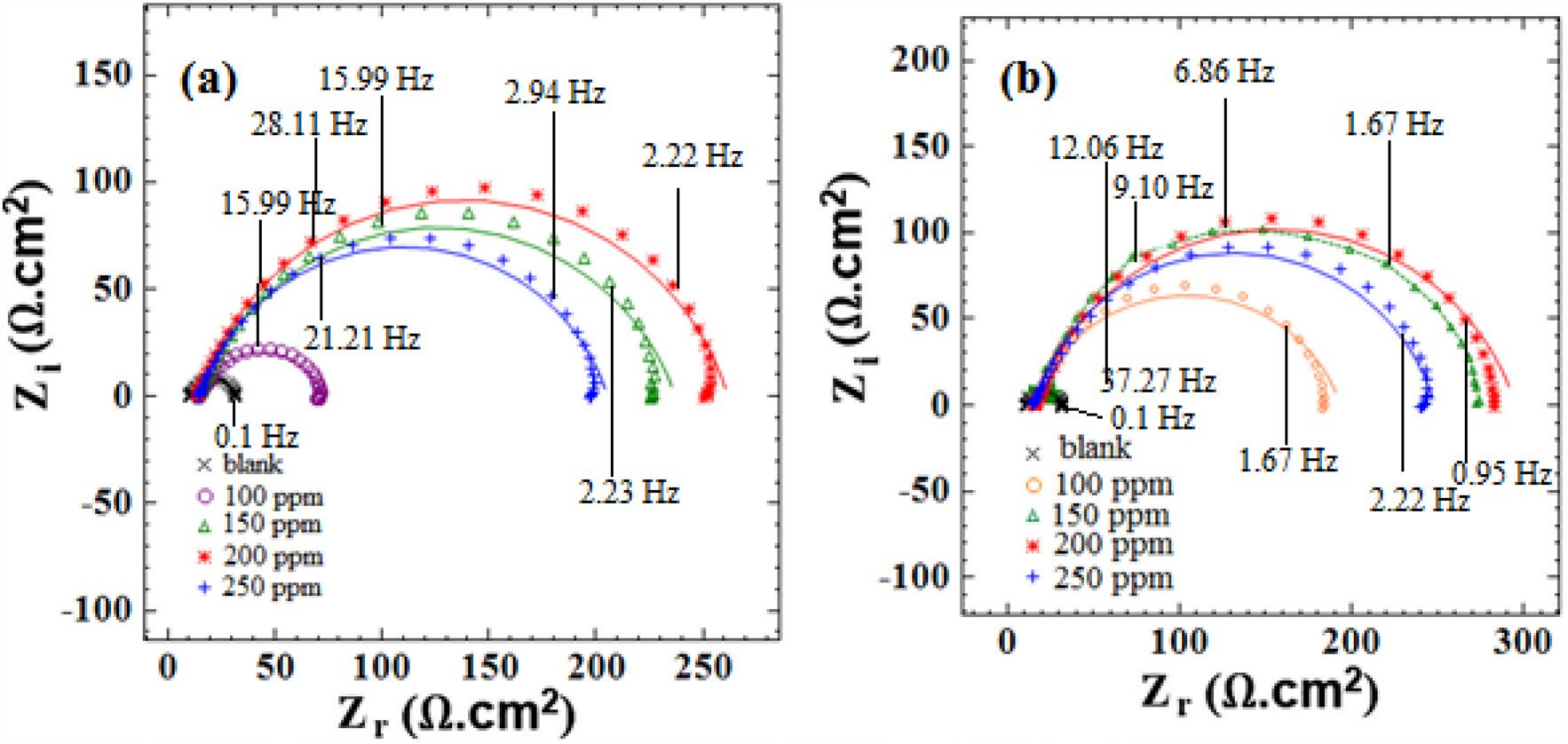
The (**a**, **b**) Nyquist plots for st-37 with different concentrations of *Viola* based on bulk, and nano size, in 0.5 M H_3_PO_4_.

*Viola* extract various inhibitory concentrations on mild steel were considered to perform EIS tests in both acidic media and examine the adsorption mechanism, whose resulting Nyquist plots are shown in Figs. 6 and 7. The inhibitor addition to hydrochloric and phosphoric acid in respective concentrations of 150 ppm and 200ppm increased the resulting Nyquist plots' diameter, subsequently increasing the charge transfer resistance (R_ct_). The impedance difference and low frequencies led to different R_ct_ values. The following equation is utilized to calculate the inhibition efficiency^43^:

2 $${IE\% }=\left(\frac{{\text{R}}_{\text{i}}-{\text{R}}_{\text{o}}}{{\text{R}}_{\text{i}}}\right)\times 100$$

which R_o_ and R_i_ represent the charge transfer resistance in an acid solution when the inhibitor absents and present, respectively.

**Figure 8:**
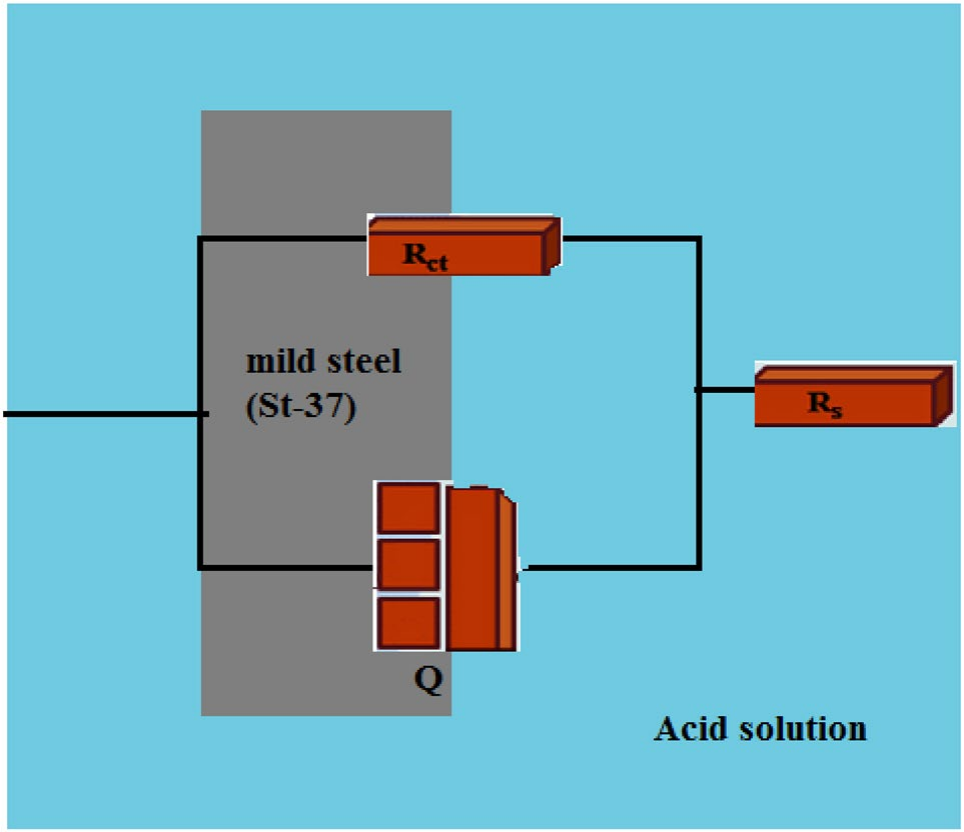
Equivalent circuit to estimate impedance diagrams.

The plot simulation and evaluation concerning the equivalent circuit represented in Fig. 8 were conducted for more accurate electrochemical impedance plot analysis. R_s_ and R_ct_ show the solution and charge transfer resistances, respectively. The below formula is used to express the constant phase element (CPE) impedance^44^:

3 $${\text{Z}}_{{{\text{CPE}}}} =A^{{ - {1}}} \left( {{\text{i}}\omega \, } \right)^{{ - {\text{n}}}}$$

In which, A, ω, and n represent the proportion coefficient, the angular frequency, and the coefficient of surface roughness, respectively, and i^2^ = − 1. CPE will have a similar function to a pure capacitor in the case of n = 1.

The below formula is used to obtain double-layer capacitance^45^:

4 $$\text{Cdl }=A {({\upomega }_{\text{max}})}^{n-1}$$

C_dl_ and ω_max_ are the respective symbols for the double layer capacitance and the maximum frequency resulting from the Nyquist plot for each analysis.

Tables 3 and 4 display the properties of the alloy when treated with various concentrations of *Viola* extract (in both bulk and nanosize forms) in the acidic media. This includes measures for instance the accuracy of the data (chi-square), the capacity of the protective layer on the surface (C_dl_), charge transfer resistance (R_ct_), and the inhibition efficiency (IEI%).

**Table 3:** Corrosion parameters derived from Nyquist curves for st-37 in HCl solution in the absence, and presence of different concentrations of inhibitor based on bulk, and nano size, at 25 $\pm$ 1 °C.

C/ppm	Chi-square	R_ct_/Ω cm^2^	n	10^6^A/F cm^−2^ s^n−1^	C_dl_/μF cm^−2^	IE%
(a) Viola/ HCl (1M)						
Blank	0.037	12.8	0.84	903	405	–
100	0.048	40.4	0.78	817	314	68
125	0.040	52.5	0.81	521	231	75
150	0.017	65.5	0.84	260	118	80
175	0.024	38	0.83	507	236	66
(b) Viola(nano)/HCl(1M)						
Blank	0.037	12.8	0.84	903	405	–
75	0.023	204	0.77	170	65	93
100	0.010	330	0.82	109	54	96
125	0.004	360	0.82	110	57	96
150	0.027	372	0.81	110	53.8	97

**Table 4:** Corrosion parameters derived from Nyquist curves for st-37 in H_3_PO_4_ solution in the absence, and presence of different concentrations of inhibitor based on bulk, and nano size, at 25 $\pm$ 1 °C.

C/ppm	Chi-square	R_ct_/Ω cm^2^	n	10^6^A/F cm^−2^ s^n−1^	C_dl_/μF cm^−2^	IE%
(a) Viola/H_3_PO_4_ (0.5M)						
Blank	0.036	21.6	0.80	573	200	–
100	0.057	58.4	0.79	470	192	63
150	0.080	222	0.79	160	68	90
200	0.042	247	0.81	107	48	91
250	0.035	190	0.81	169	78	88
(b) Viola(nano)/H_3_PO_4_ (0.5M)						
Blank	0.036	21.6	0.80	573	200	…
100	0.18	179	0.79	379	182	87
150	0.023	257	0.91	151`	150	91
200	0.11	270	0.80	229	117	93
250	0.12	233	0.83	117	76.6	91

The half-circles also demonstrate that the percentage of *IEI*% increases when there is more inhibitor. Researchers have found that smaller particles have a higher IEI% compared to larger particles of the same substance in both acid solutions.

Furthermore, when there is more *Viola*, R_ct_ increases. This is because of more extract adsorption on the steel surface, which helps protect against the corrosive media^46^. Additionally, the inhibitor provides better shielding against ions causing corrosion. When there is a lot of inhibitor in the solution (up to 150 ppm for 1. 0 M HCl and up to 200 ppm for 0. 5 M H_3_PO_4_), the values for R_ct_ and IEI% are the highest, reaching 80%, 96%, 91%, and 93% respectively. This increase demonstrates that the inhibitor creates a protective layer on the surface of the alloy, which helps stop corrosion. The rate of a chemical reaction decreases when added more *Viola* extract because it removes the inhibitor from the surface of the metal. As the concentration of the extract increased, the electric double-layer capacitor, became weaker. This might be because the constant for the electric double layer decreased^47^. In this situation, the inhibitor molecules stuck to the surface and took place with the water molecules that were originally there. The C_dl_ decreased because the inhibitor concentration increased because the inhibitor molecules had a weaker electrical field compared to water molecules, causing them to be less organized in the layer between the two materials^48^. Researchers found that the extract can create a protective layer on steel to stop corrosion, showing that *Viola* extract is effective in preventing corrosion on the alloy.

### Adsorption isotherm

Various factors, including the type of material, surface charges, corrosive conditions, ambient pH, concentration of inhibitor, distribution of charges on the inhibitor, and functional groups on the inhibitory molecule, all affect the inhibitors’ adsorption mechanism^49^. There are typically two adsorption types, one of which (physical) needs a charged metal area and charged species in the solution. The second type (chemical) is characterized by electron sharing or transfer between the inhibitor species, requiring an inhibitor with a lone pair or a free electron and an empty orbital metal^50^.

One of the main applications of adsorption isotherm in solid–liquid systems is investigating the inhibitor impacts. Here, adsorption compounds are related to the inhibitor on the surface and the soluble mass. The inhibition strength can be examined on the alloy surface. Various isotherms, including Langmuir, Temkin, and Freundlich, were reviewed to evaluate the adsorption isotherm in both acidic media. This indicates indicating the best confirmation by Langmuir adsorption isotherm for the inhibitor in both media. The equation below is used to draw Langmuir adsorption isotherm plots^51^:

5 $$\frac{\text{C}}{\uptheta }=\frac{1}{{\text{K}}_{\text{ads}}}+\text{C}$$

which, C, K_ads_, and θ are the inhibitory concentration, the adsorption equilibrium constant, and the surface coating resulting from the formula below utilizing the inhibitory efficiency, as the potentiodynamic polarization plot outcome:

6 $$\theta \, = \, \left( {{\text{IE}}\% } \right)/{1}00$$

**Figure 9:**
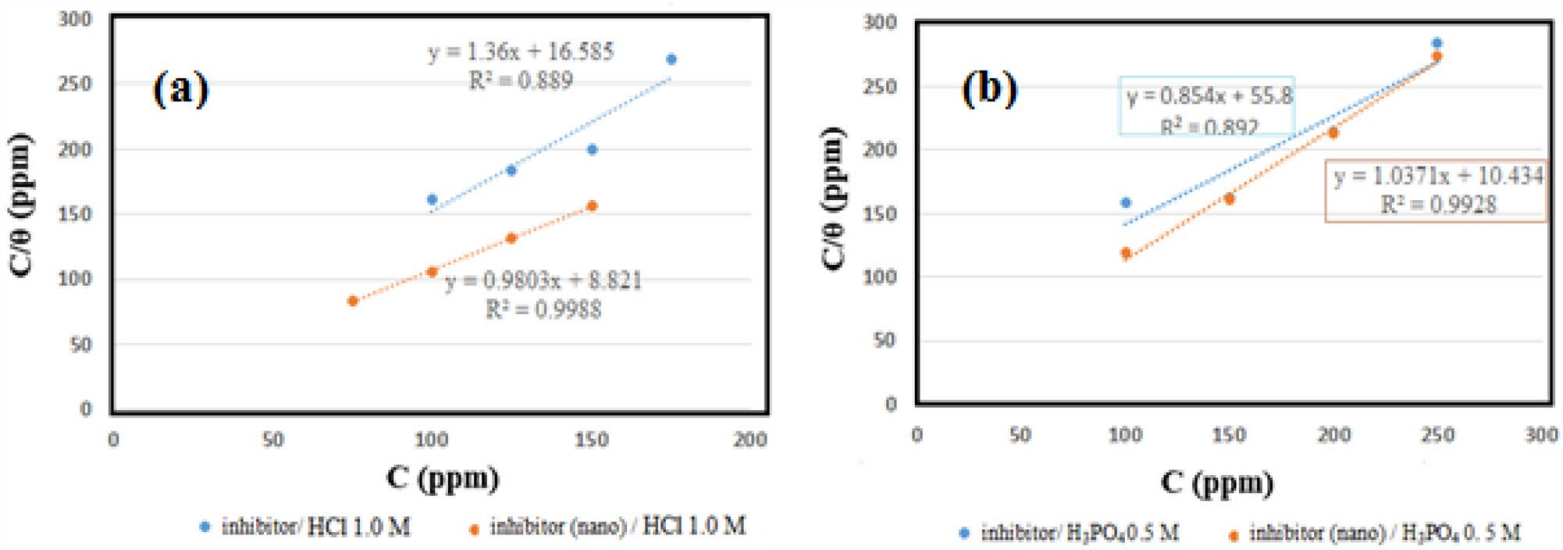
Langmuir adsorption isotherm of the inhibitor determined by Tafel polarization data for st-37 in (**a**) 1.0 M HCl solutions, and (**b**) at 0.5 M H_3_PO_4_ 25 $\pm$ 1 °C.

Hence, considering C/θ plot in C (Eq. 5), a straight line can be achieved, depicting the inhibitor obedience from Langmuir adsorption isotherm in such acidic conditions (Fig. 9).

### Impact of temperature

**Figure 10:**
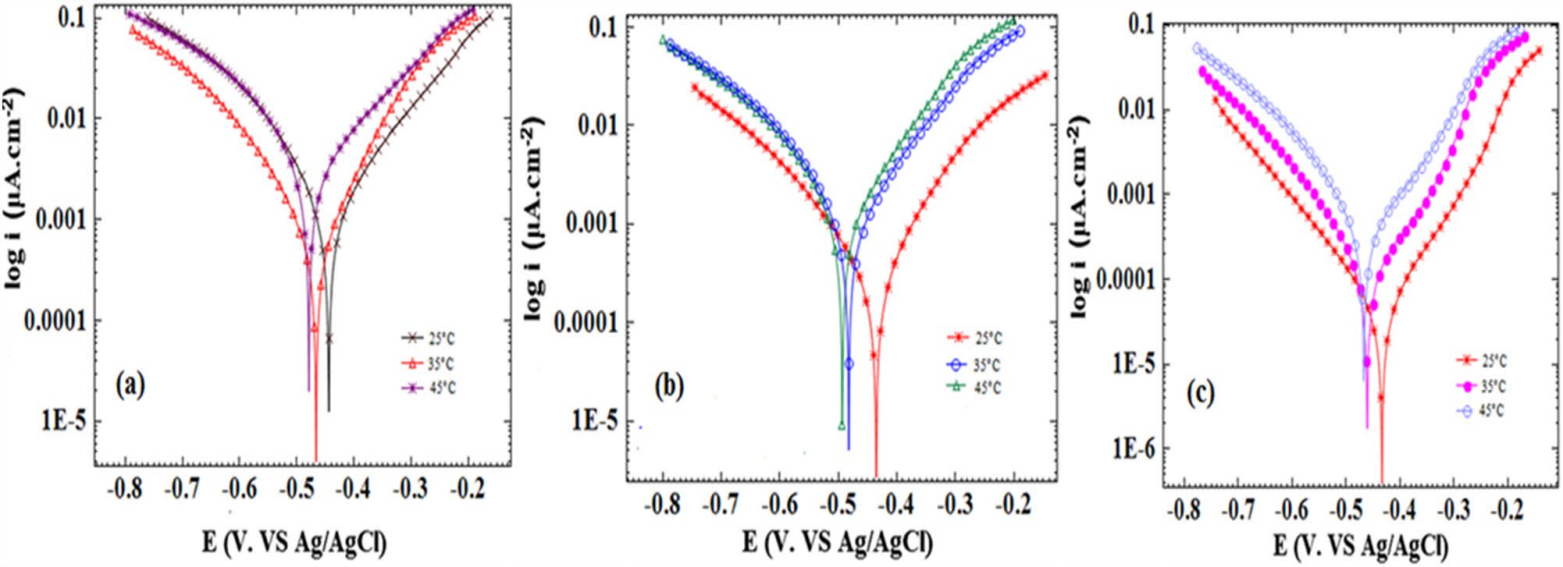
Effect of temperature on the polarization curves in 1 M hydrochloric acid solution (**a**) without inhibitor (**b**) with 150 ppm of inhibitor (**c**) with 150 ppm of inhibitor (nano).

**Figure 11:**
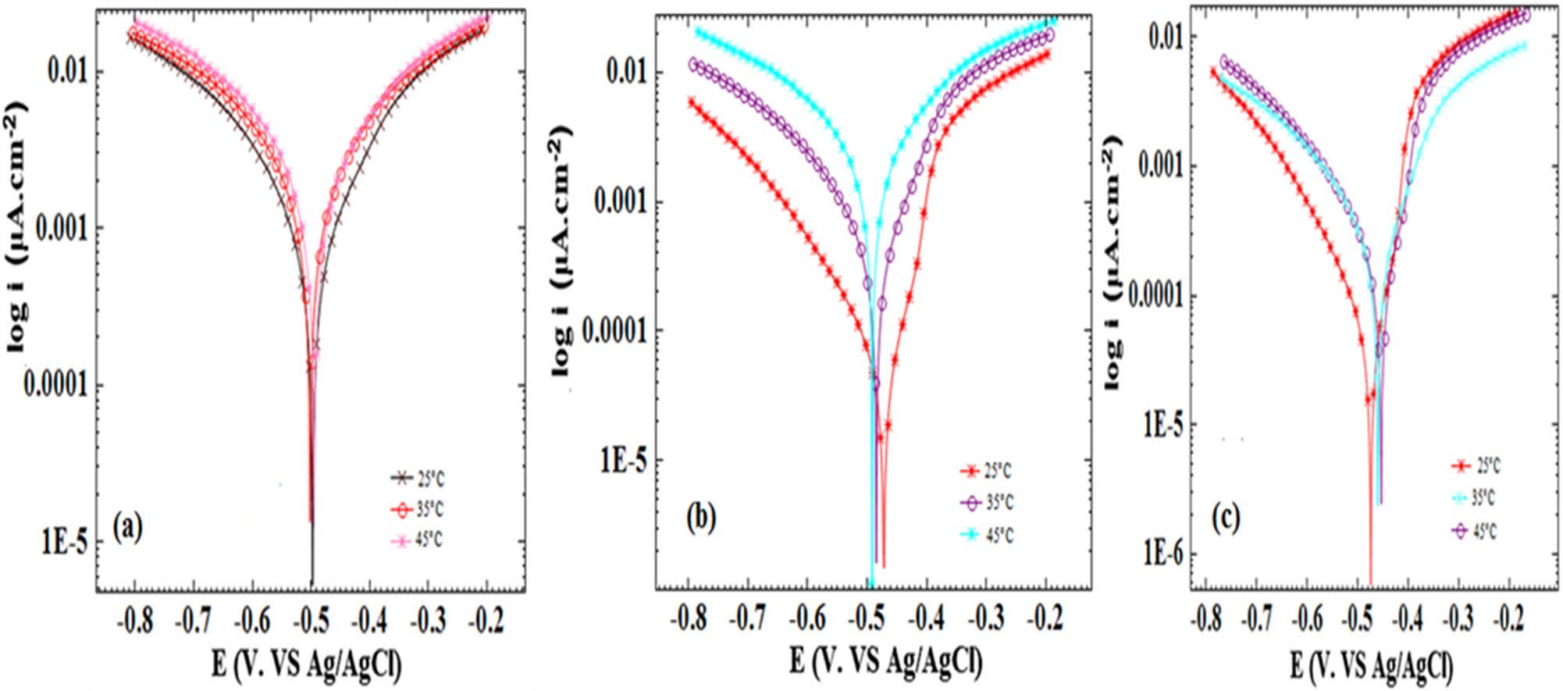
Effect of temperature on the polarization curves in 0.5 M phosphoric acid solution (**a**) without inhibitor (**b**) with 200 ppm of inhibitor (**c**) with 200 ppm of inhibitor (nano).

The extract's optimal concentration was considered to examine the temperature impact on various parameters, including corrosion current, corrosion potential, surface coating, and inhibitory percentage in the range of 25–45 °C. Figures 10 and 11 highlight the polarization plots, while Tables 5 and 6 summarize the parameters associated with the evaluation of the temperature impact.

**Table 5:** Corrosion parameters obtained from polarization measurements in 1 M HCl (a) without *inhibitor* (b) with 150 ppm of *inhibitor c)* with 150 ppm of *inhibitor (nano)* at different temperatures.

Temperature/°C	j_corr_/μA cm^−2^	− E_corr_/mV	IE%
(a) HCl without inhibitor			
25	847	444	–
35	1462	466	–
45	1701	479	–
(b) HCl with 150 ppm of inhibitor			
25	216	436	74
35	689	483	53
45	893	494	47
(c) HCl with 150 ppm of inhibitor (nano)			
25	34	434	96
35	88	462	94
45	281	468	83

**Table 6:** Corrosion parameters obtained from polarization measurements in 0.5 M H_3_PO_4_ (a) without *inhibitor* (b) with 200 ppm of *inhibitor* *(*c) with 200 ppm of *inhibitor (nano)* at different temperatures.

Temperature/°C	j_corr_/μA cm^−2^	− E_corr_/mV	IE%
(a) H_3_PO_4_ without inhibitor			
25	424	498	
35	837	502	
45	1027	495	
(b) H_3_PO_4_ with 200 ppm of inhibitor			
25	28	473	93
35	177	485	79
45	375	492	63
(c) H_3_PO_4_ with 200 ppm of inhibitor (nano)			
25	25	475	94
35	128	461	85
45	423	462	59

Given various processes resulting from the increasing temperature, its effects on the metal–acid inhibition are significantly complicated, including instances such as the inhibitor adsorption on the metal surface or decomposition under increased temperatures. The oxidation rate and reduction reactions on the metal surface are steads up under rising temperatures, preventing the uniform film formation by the inhibitor and resulting in the corrosive ions' access to the alloy surface, subsequently increasing the rate of corrosion^50^. An interesting point is the physical adsorption of inhibitory molecules on the metal surface due to the inhibition reduction at higher temperatures. On the other hand, chemical adsorption would be characterized by an opposite behaviour at higher temperatures. Thus, these two media represent a primarily physical adsorption on the alloy surface since higher temperatures have decreased the inhibition of the alloy in hydrochloric and phosphoric acid^50^.

In general, the decline in inhibition efficiency as temperature rises can be due to the diminishing time gap between desorption and adsorption of inhibitor molecules on the metal surface^52^. Consequently, the metal surface is subjected to the acidic medium for a shorter duration at higher temperatures, leading to an accelerated corrosion rate. Consequently, the percentage of inhibition efficiency declines at elevated temperatures.

For the assessment of the durability of the examined inhibitors across the experimental temperature range, the ΔIE (%) values for 150 ppm bulk and nano-sized extract in 1.0 M HCl, and 200 ppm bulk and nano-sized extract in 0.5 M H_3_PO_4_ are computed as outlined below^53^.

$$\Delta {\text{IE }}\left( \% \right) = {\text{IE25}}{-}{\text{IE45}}$$

The ΔIE (%) value indicates the general reduction in inhibition efficiency with raising the temperature from 25 to 45 °C. Smaller ΔIE (%) values suggest greater stability of inhibitor with rising temperature, leading to stronger inhibitor adsorption on the surface. The calculated ΔIE (%) values are presented in Table 7. The nano size of the extract in 1.0 M HCl exhibited greater stability with increasing temperature, and under these conditions, the extract’s nano size was the most effective inhibitor in 1.0 M HCl. The inhibition efficiency of the nano size of the extract in 1.0 M HCl demonstrated minimal change with temperature rise, suggesting a stronger adsorption bond of the nano size of the extract in 1.0 M HCl on the surface. It proves the chemisorption of the extract’s nano size in 1.0 M HCl. Hence, it is inferred that both physisorption and chemisorption are involved. Conversely, the inhibition efficiency of the bulk size of the extract in 1.0 M HCl and both sizes of the extract (bulk and nano) in 0.5 M H_3_PO_4_ exhibited a significant change with increasing temperature, indicating primarily physical adsorption.

**Table 7:** ΔIE (25–45) % values for 150 ppm bulk and nano size of the extract in 1.0 M HCl and 200 ppm bulk and nano size of the extract in 0.5 M H_3_PO_4_.

	ΔIE (25–45)%
HCl with 150 ppm of inhibitor	27
HCl with 150 ppm of inhibitor (nano)	13
H_3_PO_4_ with 200 ppm of inhibitor	30
H_3_PO_4_ with 200 ppm of inhibitor (nano)	35

The corrosion rate dependence on the temperature is shown by the Arrhenius relation^54^:

7 $${\text{j}}_{\text{corr }}=\text{ A exp}\left(\frac{{-\text{E}}_{\text{a}}}{\text{RT}}\right)$$

**Figure 12:**
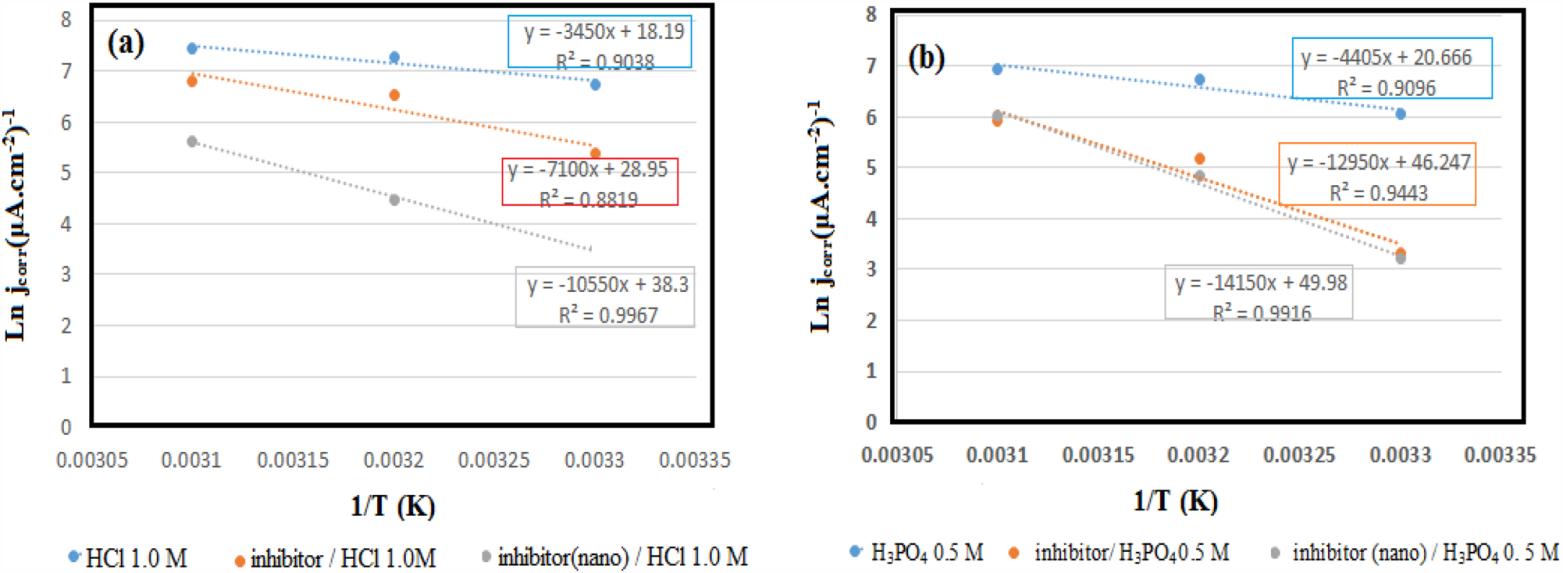
Arrhenius slopes calculated from corrosion current density for mild steel in (**a**) 1.0 M HCl solutions, and (**b**) 0.5 M H_3_PO_4_ at 25 $\pm$ 1 °C.

The corrosion current density, the frequency factor, the metal dissolution reaction's activation energy, the gas constant (8.314 J K^−1^ mol^−1^), and the absolute temperature (K) are indicated by j_corr_, A, E_a_, R, and T, respectively. As shown in Fig. 12, the slope of the Ln j_corr_ plot versus 1/T was used to measure the activation energy. Table 8 indicates the inhibitor’s estimated activation energies, highlighting an increase in the activation energy of the mild steel's corrosion reaction by the inhibitor addition to the solutions.

**Table 8:** Kinetic and Thermodynamic parameters for adsorption of inhibitor in (a) 1.0 M HCl, and (b) 0.5 M H_3_PO_4_ solutions at 25 $\pm$ 1 °C.

	E_a_ (kJ mol^−1^)	${\Delta \text{H}}_{\text{ ads}}^{\text{o}}$(kJ mol^−1^)	${\Delta \text{G}}_{\text{ ads}}^{\text{o}}$(kJ mol^−1^)	${\Delta \text{S}}_{\text{ ads}}^{\text{o}}$(kJ mol^−1^ K^−1^)
HCl	29			
H_3_PO_4_	33			
Viola/HCl	59.0	− 48.2	− 27.2	− 0.07
Viola (nano)/HCl	87.7	− 56.5	− 28.7	− 0.09
Viola/H_3_PO_4_	107.6	− 85.2	− 24.1	− 0.20
Viola(nano)/H_3_PO_4_	117.6	− 85.2	− 28.4	− 0.19

### Thermodynamic parameters

When the Langmuir adsorption isotherms are plotted for the inhibitor in the experimental solutions, a graph intercept of 1/K_ads_ is obtained, in which the adsorption equilibrium’s constant is represented by K_ads._

Calculations of the free adsorption energy values followed the K_ads_ measurement utilizing the following equation:

8 $${\Delta \text{G}}_{\text{ ads}}^{\text{o}}=-\text{RTln}\left(1\times {10}^{6}{\text{K}}_{\text{ads}}\right)$$

Table 4 indicates the ∆G˚_(ads)_ values for the inhibitor in both conditions,

Besides, ∆H˚_ads_ is determined using the equation below ^55^:

9 $$\frac{\uptheta }{1-\uptheta }=\text{ACexp}\left(-\frac{{\Delta \text{H}}_{\text{ ads}}^{\text{o}}}{\text{RT}}\right)$$

In which, T, A, C, R, ∆H^o^_ads_, and θ represent the absolute temperature in kelvins, constant frequency, the inhibitor concentration, the gas constant, the adsorption heat, and the surface coating created by the inhibitor molecules, respectively.

**Figure 13:**
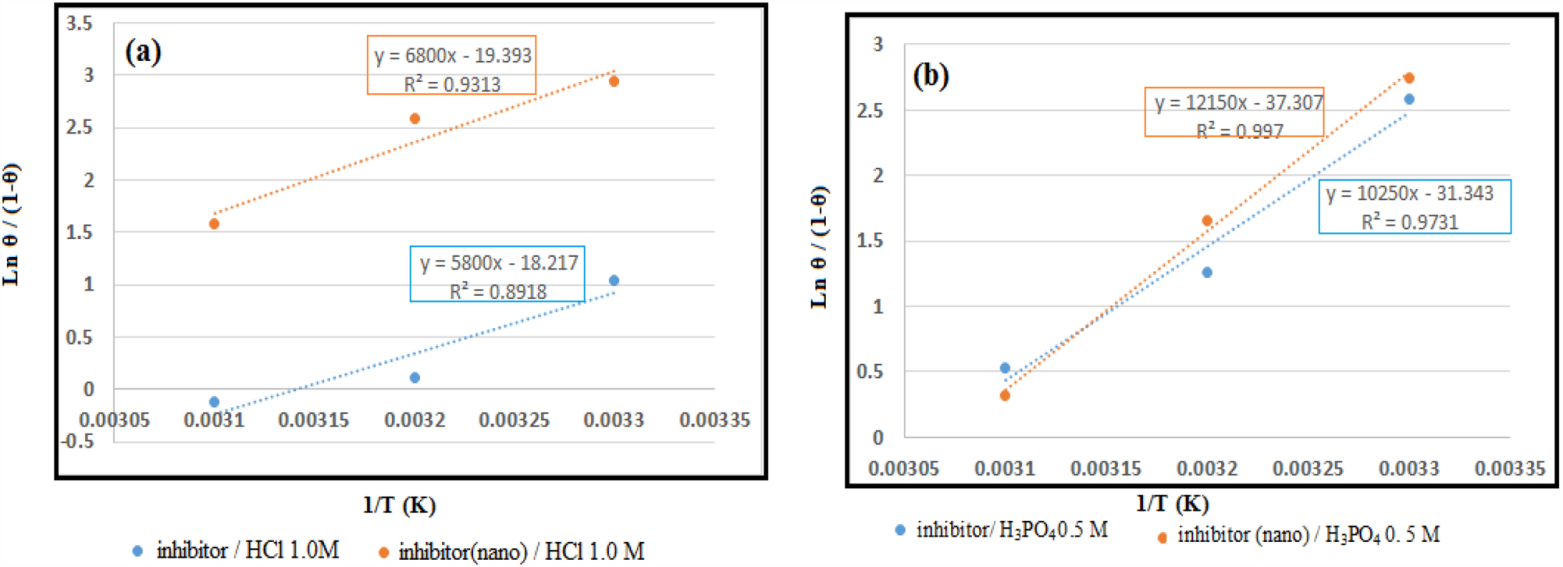
Plots of Ln (θ/1-θ) versus 1/T for mild steel in (**a**) hydrochloric acid solution containing 150 ppm of inhibitor and (**b**) phosphoric acid solution containing 200ppm of inhibitor, at different temperatures.

Figure 13 shows the plotting of Ln_(θ/(1−θ)_ versus 1/T for the greater inhibitor concentration in both solutions. Besides, (− ∆H^o^_ads)_)/R) given the slope of the graph's linear parts, utilized for the ∆H^o^_(ads)_ measurement.

Besides, ${\Delta \text{S}}_{\text{ ads}}^{\text{o}}$ is measured using Eq. 10^56^:

10 $${\Delta \text{G}}_{\text{ ads}}^{\text{o}}={\Delta \text{H}}_{\text{ ads}}^{\text{o}}-\text{T}{\Delta \text{S}}_{\text{ ads}}^{\text{o}}$$

Table 8 illustrates the values of measured thermodynamic and kinetic factors.

As suggested by the $\text{activation energy }\left({E}_{a}\right)$ values in the inhibitor's absence and presence, there was an increase in the activation energy with the inhibitor. The increased corrosion activation energy with the inhibitor highlights close associations of inhibition and adsorption^57^. The values of K_ads_ can be calculated to determine the ${\Delta \text{G}}_{\text{ ads}}^{\text{o}}$ values, indicating the substance adsorption on the surface. Two ways for substance adsorption when examining corrosion prevention include physisorption and chemisorption^58^. More simply, physical adsorption displays the standard adsorption free energy values up to − 20 kJ/mol. However, values of < − 40 kJ/mol are obtained in the case of chemical adsorption^1^.

The evaluated free adsorption energy (${\Delta \text{G}}_{\text{ ads}}^{\text{o}})$ for the inhibitor in two acidic media with the extract's bulk and nano size was less than − 40 kJ mol^−1^, ranging between the physical and chemical adsorptions. Interestingly, the adsorption phenomenon is not displayed merely for a molecule regarded as a complete form of physical or chemical adsorption phenomena. Several molecules may show adsorption behaviours with occasionally overcoming chemical or physical adsorption types^59^. The spontaneous process is confirmed by the negative value of the free adsorption energy (${\Delta \text{G}}_{\text{ ads}}^{\text{o}})$ displayed for the inhibitor^60^. The adsorption enthalpy (${\Delta \text{H}}_{\text{ ads}}^{\text{o}})$ negative values for the inhibitor (bulk and nano size) confirm the exothermic process for the inhibitor's adsorption on the alloy for both media. The decreased Entropy was highlighted by the small and negative adsorption entropy (${\Delta \text{S}}_{\text{ ads}}^{\text{o}})$ values in the hydrochloride and phosphoric acid solution comprising bulk and nano size of inhibitor.

Table 9 provides a comparison between the current study and other research endeavors utilizing plant extracts as corrosion inhibitors in acidic environments. It is inferred that *Viola* extract, particularly in nanometer and bulk sizes, emerges as a promising option for enhancing the corrosion resistance of mild steel alloy in both 1.0 M HCl and 0.5 M H_3_PO_4_ solutions.

**Table 9:** Summary of similar studies for plant extract as a corrosion inhibitor and in comparison to this study.

References	Inhibitor	Concentration of inhibitor	alloy	Corrosive medium	PCE (%)
^61^	*African mangosteen leaves extract*	1.5 g/L	Carbon steel	0.5 M H_2_SO_4_	95.57
^28^	*Ammi visnaga* extract	700 ppm	Carbon steel	1.0 M HCl	84
^29^	*Quince seed* extract	800 ppm	Mild steel	1.0 M HCl	95
^30^	*Inula viscosa* leaves extract	600 ppm	Carbon steel	1.0 M HCl	92
^62^	*Dolichandra unguis-cati leaves extract*	0.76 g/L	Mild steel	1.0 M HCl	93.61
^31^	*Eucalyptus leaf extract*	0.2 mol/L	Mild steel	0.5 M H3PO4	78
^33^	*the essential oil of Artemisia*	1 g/L	Stainless steel	1 M H3PO4	85.2
This work	*Viola* extract	150 ppm	Mild steel	1.0 M HCl	80
This work	*Viola* extract (nano)	150 ppm	Mild steel	1.0 M HCl	96
This work	*Viola* extract	200 ppm	Mild steel	0.5 M H3PO4	91
This work	*Viola* extract (nano)	200 ppm	Mild steel	0.5 M H3PO4	93

### Surface morphology examination

**Figure 14:**
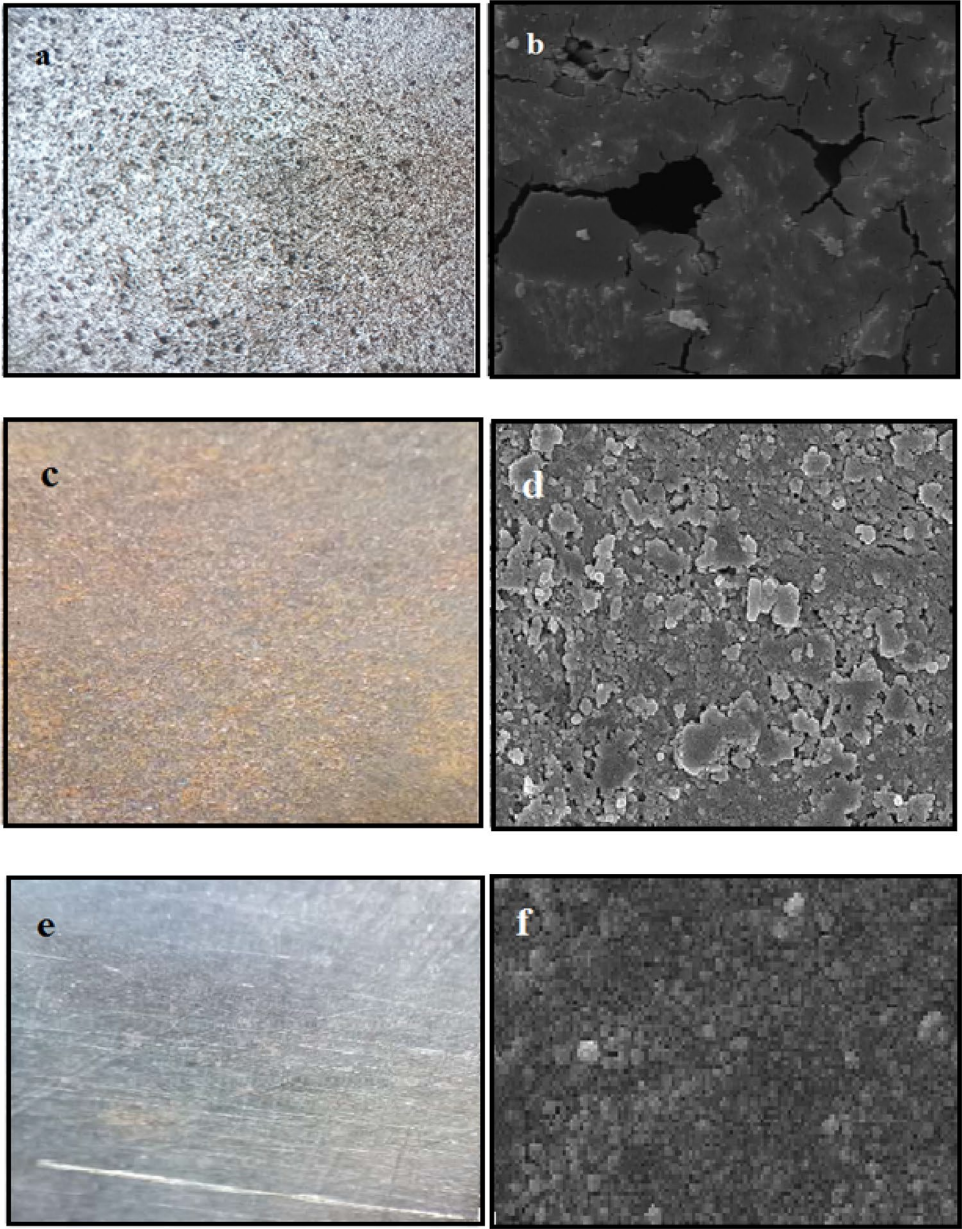
The images of the st-37 surface after 24 h immersion in 1.0 M HCl solution in the (**a**, **b**) absence, (**c**, **d**) presence of 150 ppm of *Viola* extract in bulk size (**e**, **f**), and presence of 150 ppm of *Viola* extract in nano size using optical, and Scanning electron microscopy, respectively.

**Figure 15:**
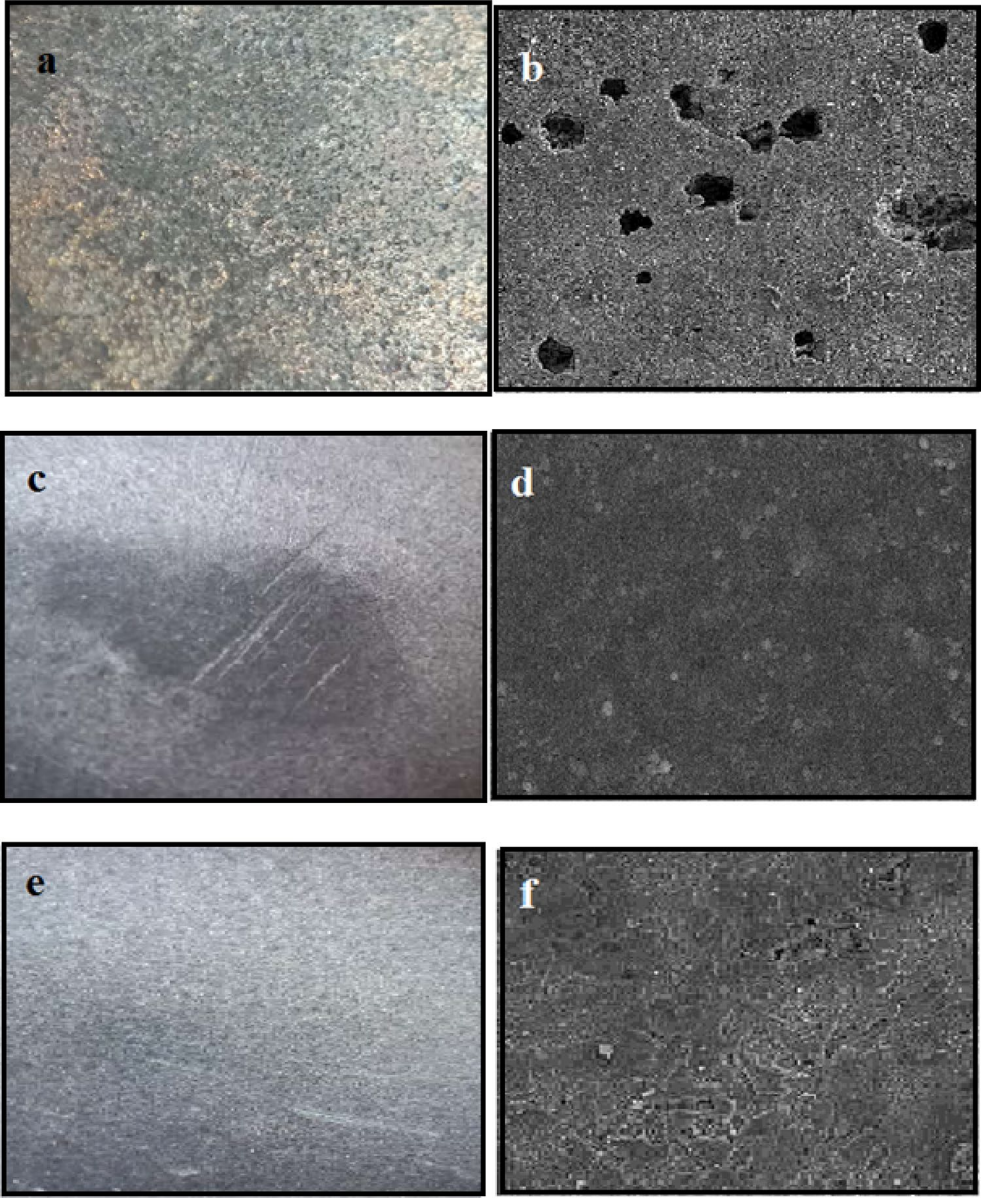
The images of the st-37 surface after 24 h immersion in 0.5 M H_3_PO_4_ solution in the (**a**, **b**) absence(**c**, **d**) presence of 200 ppm of *Viola* extract in bulk size (**e**, **f**), and presence of 200 ppm of *Viola* extract in nano size, using optical microscopic.

Figures 14 and 15 represent the optical and Scanning electron microscopy of the alloy surface plunged in 1 M HCl and 0.5 M H_3_PO_4_ solutions when 150 and 200 ppm of extract was lacking and present for 24 h. As shown in Figs. 14a, b and 15a, b, surface corrosion was evident when the inhibitor was absent, leading to several pits. On the other hand, Figs. 14c–f and 15c–findicate the formation of somehow a protective layer on the surface of the steel as the inhibitor was added, generating a block on the surface of the metal, preventing the corrosive ions from reaching the metal surface, and passivating the active sites on the surface of the alloy. Accordingly, the inhibitor function on the alloy surface is illustrated, including prevention of the metal surface corrosion by creating the protective layer.

## Conclusions

The impact of *Viola* extract, whether in bulk or nanometer size, as a corrosion inhibitor for mild steel in 1.0 M HCl and 0.5 M H_3_PO_4_ solutions was examined.

1. The findings from PP and EIS curves reveal that the inhibition efficiency increased with the rise in extract concentration up to a certain threshold.
2. By impedance spectroscopy method, the nano extract showed the highest *IE*% of 97% in HCl media, while the bulk extract had a highest *IE*% of 80% at 150 ppm concentration. The highest *IE*% of the nano and bulk extracts equaled 93% and 91% at 200 ppm concentration in the H_3_PO_4_ solution. Interestingly, there was good agreement between the polarization and EIS measurement results.
3. Based on the PP measurements, *Viola* extract (bulk and nano size) could perform as a mixed inhibitor in both acid solutions.
4. The computed values of enthalpy and free energy of adsorption revealed that physical and chemical adsorption occurred on the surface of st-37, after the Langmuir isotherm. Additionally, the negative value of adsorption free energy suggests that inhibitor molecules spontaneously adsorb onto the metal surface. Specifically, the adsorption of the nano size of the extract in 1.0 M HCl was predominantly chemisorption. In comparison the bulk size of the extract in 1.0 M HCl and both sizes of the extract (bulk and nano) in 0.5 M H_3_PO_4_ primarily underwent physisorption.
5. SEM and optical techniques were employed to validate the corrosion assessments. Consequently, a uniform and minimally disturbed surface was observed with the ideal concentration of *Viola* extract in acidic solutions. This led to the manifestation of corrosion inhibition efficacy and the formation of a protective inhibitor film.

In conclusion, in comparison to findings from other researchers, it is inferred that *Viola* extract exhibits the smallest optimal concentration yet maintains effective performance. Thus, utilizing *Viola* extract in nanometer size allows for a substantial reduction in the required inhibitor concentration while enhancing corrosion resistance and efficiency. This presents an economical, environmentally friendly, and effective approach to mitigating mild steel corrosion in an acidic medium. Therefore, *Viola* extract emerges as a promising candidate for enhancing the corrosion resistance of mild steel alloy in 0.5 M H_3_PO_4_ and 1.0 M HCl solutions.

## Data Availability

The datasets used and/or analysed during the current study available from the corresponding author on reasonable request.
